# Genetic Polymorphisms Associated with Prothrombin Time and Activated Partial Thromboplastin Time in Chinese Healthy Population

**DOI:** 10.3390/genes13101867

**Published:** 2022-10-15

**Authors:** Fan Zhang, Guangyan Mu, Zhiyan Liu, Qiufen Xie, Hanxu Zhang, Shuang Zhou, Zhe Wang, Kun Hu, Zining Wang, Xia Zhao, Yimin Cui, Qian Xiang

**Affiliations:** 1 Department of Pharmacy, Peking University First Hospital, Beijing 100034, China; 2 School of Pharmaceutical Sciences, Peking University Health Science Center, Beijing 100191, China; 3 Institute of Clinical Pharmacology, Peking University, Beijing 100191, China

**Keywords:** activated partial thromboplastin time (APTT), genome-wide association analysis, healthy population, prothrombin time (PT), whole-exome sequencing

## Abstract

(1) Background: The purpose of this study was to evaluate the effect of gene polymorphisms on prothrombin time (PT) and activated partial thromboplastin time (APTT) in a healthy Chinese population. (2) Methods: A total of 403 healthy volunteers from a series of novel oral anticoagulants (NOACs) bioequivalence trials in China were included. Coagulation tests for PT and APTT were performed in the central lab at Peking University First Hospital. Whole-exome sequencing (WES) and genome-wide association analysis were performed. (3) Results: In the correlation analysis of PT, 105 SNPs from 84 genes reached the genome-wide significance threshold (*p* < 1 × 10^−5^). Zinc Finger Protein 594 (*ZNF594*) rs184838268 (*p* = 4.50 × 10^−19^) was most significantly related to PT, and Actinin Alpha 1 (*ACTN1*) was found to interact most with other candidate genes. Significant associations with previously reported candidate genes Aurora Kinase B (*AURKB*), Complement C5(*C5*), Clock Circadian Regulator (*CLOCK*), and Histone Deacetylase 9(*HDAC9*) were detected in our dataset (*p* < 1 × 10^−5^). PiggyBac Transposable Element Derived 2(*PGBD2*) rs75935520 (*p* = 4.49 × 10^−6^), Bromodomain Adjacent To Zinc Finger Domain 2A(*BAZ2A*) rs199970765 (*p* = 5.69 × 10^−6^) and Protogenin (*PRTG*) rs80064850 (*p* = 8.69 × 10^−6^) were significantly correlated with APTT (*p* < 1 × 10^−5^). The heritability values of PT and APTT were 0.83 and 0.64, respectively; (4) Conclusion: The PT and APTT of healthy populations are affected by genetic polymorphisms. *ZNF594* and *ACTN1* variants could be novel genetic markers of PT, while *PRTG* polymorphisms might be associated with APTT levels. The findings could be attributed to ethnic differences, and need further investigation.

## 1. Introduction

Prothrombin time (PT) and activated partial thromboplastin time (APTT) are commonly used parameters for screening coagulation disorders and monitoring anticoagulant therapy [1,2]. APTT is widely used in the screening of inherited and acquired coagulation factor deficiencies in intrinsic and common coagulation pathways, while the PT test is more sensitive for extrinsic coagulation system function [3,4]. In addition, PT and APTT monitoring are essential for clinical decision-making in critical situations, such as bleeding, thromboembolism, and emergency surgery, and may also predict the perioperative bleeding risk of patients taking anticoagulants undergoing elective surgery [5,6].

Except for the influence of sample preparation and detection methods, PT and APTT levels are affected by some physiological factors. It is well known that the hemostatic system exhibits dynamic age-related evolution and thus can present differences in PT and APTT at different ages [7]. In addition, genetic factors may influence PT and APTT levels. A European genome-wide association study found that *KNG1, HRG, F11, F12,* and *ABO* are associated with APTT, while *F7* and *PROCR/EDEM2* are related to PT [8,9]. A recent study also found a positive correlation between the allele load of the *JAK2V617F* mutation and APTT [10]. Polymorphisms in PT and APTT levels also vary greatly in healthy people. Some people have a low baseline level; even if the PT or APTT is doubled, it may still be within the normal range. However, some people have a high baseline level, and a slight extension (less than 3 s) will exceed the normal range. This indicates a potential impact of genetic factors on PT and APTT in healthy people. However, the overall effects of gene polymorphisms on these coagulation indexes are still poorly understood. Moreover, there is no large-scale genome-wide association analysis (GWAS) on PT and APTT levels among Asians.

In this study, we conducted whole-exome sequencing (WES) and association analysis in a healthy Chinese population to further explore genetic markers and their heritability affecting PT and APTT.

## 2. Materials and Methods

### 2.1. Study Population

The study population for genome-wide association analysis comprised a series of novel oral anticoagulants (NOACs) bioequivalence trial participants in China. Healthy participants were defined according to the general inclusion criteria of BE trials, including history, physical examination, vital signs, 12-lead electrocardiogram (ECG), ultrasound and imaging examination, laboratory examination, and alcohol exhalation, etc. A total of 424 healthy volunteers aged 18–60 years were enrolled in 10 centers. None of the participants enrolled in the study had taken any drug for at least 4 weeks before the start of the study. Blood samples were collected at baseline for genotyping and coagulation tests. All protocols and informed consent were reviewed and approved by the independent ethics committee of Peking University First Hospital and all participating centers. Before the start of the study, all subjects were informed of the purpose, duration, and potential risks of the study and provided written informed consent. This study was registered on ClinicalTrial.org with the registration number NCT03161496.

### 2.2. PT and APTT Measurement

Blood samples were collected in sodium citrate test tubes (3.2% *v*/*v*) and centrifuged at 2500× *g* at room temperature for 15 min within 60 min of sampling. Plasma samples were transferred to cryovials and stored at −70 °C. Coagulation tests for PT and APTT were performed in the central lab in Peking University First Hospital within 6 months after sampling [11]. Coagulation tests showed circadian rhythm [12], so all samples were tested at similar times during the day to avoid the values changing. PT and APTT were measured using validated Coagulation Method Assay Kits (Thromborel-S^®^ and Actin^®^, Siemens Healthcare Diagnostics Products GmbH, Marburg, Germany) following a standard protocol. The units of the PT and APTT measurement values were seconds.

### 2.3. Whole-Exome Sequencing and Quality Control

Blood samples were collected in EDTA-K2 test tubes, transferred to cryovials, and stored at <−70 °C until genotyping. WES [13,14] for all included samples was conducted at CapitalBio Technology Co., Ltd. (Beijing, China). The quality of isolated genomic DNA was assessed by using these three methods in combination: (1) DNA degradation and contamination were monitored on 0.8% agarose gels. (2) DNA purity was checked using the NanoPhotometer^®^ spectrophotometer (IMPLEN, Westlake Village, CA, USA). (3) DNA concentration was measured by Qubit^®^ DNA Assay Kit in Qubit^®^ 3.0 Flurometer (Invitrogen, Carlsbad, CA, USA). The sequencing library was prepared with the Agilent Sure SelectXT Human All Exon V6 Kit (Agilent Technologies, Palo Alto, CA, USA), and the genomic DNA was cut to an average fragment size of 200 bp. The ends of DNA fragments underwent a terminal repair process, and then the A-tail and adapter were connected. DNA fragments with adaptors attached at both ends were selectively enriched by polymerase chain reaction (PCR). Biotin-labeled probes and magnetic bead selection were used for library hybridization and exome capture. The captured library was enriched and labeled with PCR for sequencing. The final library was quantified using the Kapa Library Quantification Kit (Kapa Biosystems, Boston, MA, USASouth Africa) and Agilent 2100 Bioanalyzer. The final 2 × 150 bp paired-end sequencing was generated using Illumina NovaSeq 6000 sequencers (Illumina, San Diego, CA, USA).

Variant filtering and prediction were performed on 173,128 SNPs using PLINK v 1.9 [15]. SNPs with a missing rate > 10%, minor allele frequency (MAF) < 0.01 or Hardy-Weinberg equilibrium (HWE) *p* < 1 × 10^−6^ were removed. Exclusion criteria for samples included individuals with genotyping rate < 0.3%, abnormal heterozygosity values and genetic outliers. Principal component analysis (PCA) was conducted for population stratification correction using PLINK v 1.9 and R package 4.2.0 [16] (Supplementary Figure S1). A total of 101,844 SNPs in 403 samples were included for subsequent analysis according to the quality control criteria.

### 2.4. Data Analysis and Functional Annotation

Linear regression of PT or APTT in an additive genetic model was conducted to determine the association between SNPs and phenotypes, adjusting for age and sex. The association analyses were performed using Plink v 1.9. A genome-wide significance threshold *p* < 1 × 10^−5^ was used to correct for multiple testing. GWAS results were presented as Manhattan plots and QQ plots using the R package. The top hit SNPs were further illustrated as regional association plots using LocusZoom [17].

Haploreg v4.19 [18] was queried to investigate the corresponding functional annotation of all identified SNPs with genome-wide significance. Reactome Gene Sets and Gene Oncology (GO) pathway analyses were carried out in Metascape [19] to identify differentially enriched genes. The protein‒protein interaction (PPI) analysis was constructed on STRING [20]. GCTA 1.94.0 β [21] and the R package were used to calculate the heritability of single nucleotide polymorphisms at the genome-wide level using a genomic-relatedness-based restricted maximum-likelihood (GREML) model to estimate the proportion of phenotypic variation explained by single nucleotide polymorphisms [22,23].

## 3. Results

### 3.1. Baseline Characteristics and Coagulation Parameters

A total of 403 healthy people aged 18–60 years old were included in this study. The highest values of PT and APTT reached 3 times and 2 times the lowest values, respectively. The characteristics of the study participants are shown in Table 1.

### 3.2. Discovery of Candidate SNPs for PT and APTT

**Figure 1:**
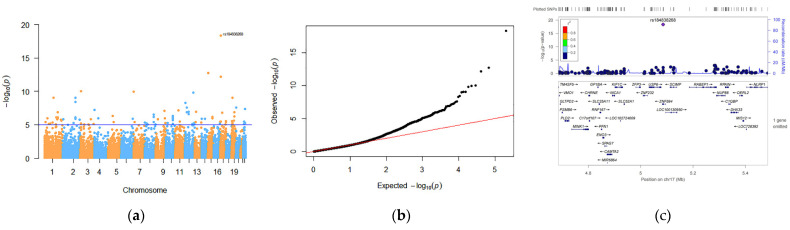
Discovery of candidate SNPs for PT: (**a**) Manhattan diagram. The horizontal line in the Manhattan diagram indicates a threshold *p* < 1 × 10^−5^. (**b**) QQ-PLOT. (**c**) Regional association plot of SNPs associated with PT. SNPs on adjacent chromosomes are separated by blue and orange dots. The highest point is SNP rs184838268.

In the PT association analysis, 105 SNPs located in 84 genes reached the preset threshold (*p* < 1 × 10^−5^). The strongest association of PT was with rs184838268 (*p* = 4.50 × 10^−19^) located in an exon in Zinc Finger Protein 594 (*ZNF594*) on chromosome 17 (Figure 1). Detailed representations of these candidate SNPs with functional annotations are listed in supplementary Tables S1 and S2.

**Figure 2:**
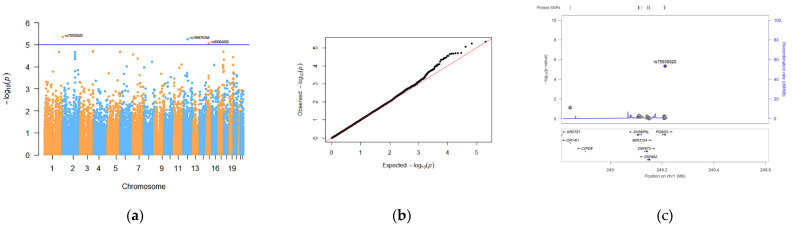
Discovery of candidate SNPs for APTT: (**a**) Manhattan diagram. The horizontal line in the Manhattan diagram indicates a threshold *p* < 1 × 10^−5^. (**b**) QQ-PLOT. (**c**) Regional association plot of SNPs associated with APTT. SNPs on adjacent chromosomes are separated by blue and orange dots. The highest point is SNP rs75935520.

In the APTT association analysis, only 3 SNPs passed the GWA significance level (*p* < 1 × 10^−5^). The rs75935520 (*p* = 4.49 × 10^−6^) located in an exon in PiggyBac Transposable Element Derived 2 (*PGBD2*) on chromosome 12 showed the strongest correlation signal with APTT (Figure 2). The other two associated SNPs were introns rs199970765 (*p* = 5.69 × 10^−6^) located in Bromodomain Adjacent To Zinc Finger Domain 2A (*BAZ2A*) on chromosome 12 and rs80064850 (*p* = 8.69 × 10^−6^) located in Protogenin (*PRTG*) on chromosome 15.

### 3.3. Pathway Enrichment and PPI

**Figure 3:**
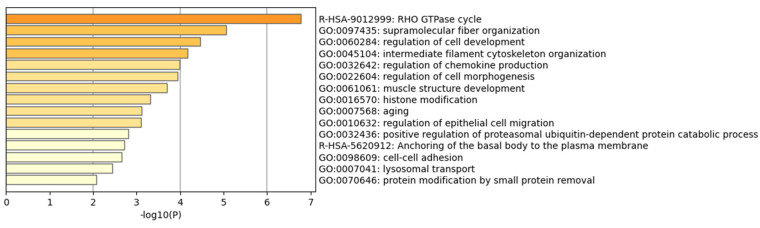
Significant Gene Ontology biological processes in PT genome-wide association analysis.

Pathway and enrichment analyses were performed from the 84 annotated candidate genes of PT, and one Reactome Gene Sets pathway and three GO pathways of significance were identified (Figure 3). A total of 15 pathways were enriched, of which the Rac1 GTPase cycle pathway was associated with the coagulation pathway. PPI analysis of these candidate genes identified 23 key node genes in the PPI network (Supplementary Figure S2). Actinin Alpha 1(*ACTN1*) was found to be a central node gene, indicating that it might have an important role in the process of human coagulation. Eleven genes were enriched in the most significant pathways, including the *ACTN1* and Aurora Kinase B (*AURKB*) genes, which were reported to be involved in coagulation pathways.

### 3.4. Heritability and Phenotypic Variance Explained

The heritability of PT and APTT are shown in Table 2. The phenotypic variation explained (PVE) of the mixed linear model (MLM) was used to estimate the genetic contribution rate of each SNP. The heritability values of PT and APTT were 0.83 and 0.64, respectively.

## 4. Discussion

This WES and GWAS evaluated the effect of gene polymorphisms on PT and APTT in a healthy Chinese population and identified several candidate variants. The large sample size and rigorous protocol design ensured the study quality.

In our study, *ZNF594* rs184838268 was found to have the strongest association with PT (*p* = 4.50 × 10^−19^). *ZNF594* is one of the zinc finger genes. The zinc finger proteins belong to a group of proteins that bind divalent zinc ions through a combination of cysteine and histidine. The functions of zinc finger proteins can include bifunctional RNA and DNA binding, transcriptional repression, single-stranded DNA binding, RNA-binding, stimulation of transcription, etc. [24]. Previous studies have suggested that the *ZNF594* gene is associated with airway remodeling in asthma, which is characterized by the thickening of the reticular basement membrane (RBM) and regulated through DNA transcription [25]. Another study speculated that *ZNF594* might be a crucial gene in human primary bronchial epithelial cells, which could impact the effectiveness of inhaled 2-adrenoceptor agonists in managing asthma [26]. Subcutaneous angiogenesis is one of the features of asthma and an initiator of coagulation. The tissue factor (TF) is also a key player in angiogenesis. In mechanical injury, bronchial epithelial cells may be a potential source of secreted TF [27] and may respond rapidly to mechanical injury by forming a cross-linked fibrin matrix [28]. Several studies have provided evidence of platelet activation in asthma, as evidenced by an increase in platelet-derived mediators, such as platelet factor 4, beta-thromboglobulin (β-TG), RANTES, and thromboxane [29]. However, there are no reports on the *ZNF594* gene affecting the coagulation pathway, and further exploration is needed.

In our pathway and enrichment analysis, the most significant pathway was the Rac1 GTPase cycle. Rac is a Rho GTPase family member that has a crucial role in regulating platelet function by mobilizing the actin cytoskeleton during the activation of platelets [30,31,32]. Extracellular matrix proteins become exposed after vascular injury and send signals to the hemostasis system, leading to platelet cytoskeleton modification to an active state. Rac1 is required for the integrity of thrombosis aggregation. Rac1 controls actin polymerization on the membrane to encourage the growth and development of platelet lamellar pseudopodia and to stimulate platelet diffusion [32,33,34]. Aslan et al. [35] discovered that Rac1- and p21-activated kinases (PAKs) mediate thrombin-triggered platelet reactions after thrombin-activated platelets. Following Rac1 activation, the PAK signaling system contributes to platelet diffusion and aggregation by promoting thrombin-mediated activation of the MEK/ERK pathway, Akt, and calcium signaling. Nine genes from our dataset were found to be enriched in the most important Rac1 GTPase cycle pathway, among which *ACTN1* and *AURKB* were previously reported to be associated with coagulation pathways. The *ACTN1* gene was also presented as a core node in the subsequent PPI network.

*ACTN1* encodes α-actinin-1, a member of the actin cross-linking protein superfamily, which is involved in the organization of the cytoskeleton [36,37]. A genome-wide association analysis of platelet count (PLT) in 12,491 Hispanics/Latinos revealed that *ACTN1* rs117672662 was most substantially associated with PLT (*p* = 1.16 × 10^−28^). *ACTN1* was found to be correlated with hereditary thrombocytopenia [38]. A study in Japanese congenital macrothrombocytopenia (CMTP) patients revealed that *ACTN1* polymorphisms might result in a half-reduction in platelet counts and a 30% increase in platelet size [39]. Thus, the impact of *ACTN1* on platelet function deserves further research.

Aurora kinase is a serine/threonine kinase. Aurora members have an important role in mitosis, and *AURKB* (Aurora kinase B) is an important Aurora member [40]. The expression and function of *AURKB* may affect the production of platelets, while its inhibition of Aurora B can induce growth arrest and apoptosis of megakaryocytes (precursors of platelets) during mitosis [41,42]. A small study in Japan showed that the gene expression level of Aurora B was related to PT. The PT of patients with high expression of the Aurora B gene in nontumor liver tissues of patients with hepatocellular carcinoma was higher than that of patients with low expression of the Aurora B gene [11.6 (11.1, 12.0) vs. 12.0 (11.5, 12.9), *p* = 0.03] [43]. A recent study also identified upregulation of *AURKB* expression in platelets among STEMI cases compared with NSTEMI cases [44]. Therefore, *AURKB* seemed to be an appropriate genetic marker for PT.

Furthermore, significant associations with previously reported candidate genes Complement C5 (*C5*), Clock Circadian Regulator (*CLOCK*), and Histone Deacetylase 9 (*HDAC9*) were detected in our GWAS of PT. *C5* rs2230212 showed some correlation with PT (7.74 × 10^−8^). Studies suggested several connections between complement cascade responses and coagulation. The blood coagulation system and the complement system are enzymatic cascades that support host defense. These two system activation mechanisms are correlated, and *C5* can be activated by coagulation enzymes, including thrombin and kallikrein [45]. In venous thrombosis, *C5* promotes thrombosis by activating tissue factor activation [46]. The C5b-9 terminal complement complex is assembled on cell membranes due to complement system activation [47]. A dose-dependent increase in the binding of the coagulation factors Va and Xa to the plasma membrane is caused by the membrane assembly of the complement proteins C5b-9 on human platelets, and this is accompanied by a notable rise in the activity of the enzyme platelet prothrombinase [48].

In the present study, *CLOCK* rs3762836 showed some correlation with PT (*p* = 1.105 × 10^−6^), and the correlation between the *CLOCK* gene and coagulation indexes has been reported in previous studies. The circadian rhythm governs the coagulation function of the cardiovascular system [49], and the *CLOCK* gene can regulate the expression of thromboproteins [50]. According to previous studies, the circulatory system’s downregulation of *CLOCK* impacts on coagulation and fibrinolytic factors. Mice with downregulated *CLOCK* gene expression were reported to have longer PT and APTT and were less likely to develop thrombosis [51]. However, some studies have found that the *CLOCK* mutation could affect the fibrinolytic system, but the coagulation parameters (APTT, PT) were not affected by the clock mutation [52]. More research is needed to understand how the *CLOCK* gene affects the coagulation system.

*HDAC9* is an important determinant of vascular smooth muscle cell phenotype and calcification, and *HDAC9* deficiency significantly reduces vascular calcification in mice [53]. Another study showed that *HDAC9* might mediate inflammatory injury in vascular endothelial cells by regulating the phosphorylation level of P38 mitogen-activated protein kinase (P38 MAPK) [54]. Previous studies have also shown that the P38 MAPK signaling pathway is an important signaling pathway in the coagulation pathway. P38 MAPK can control thrombospondin expression by regulating RNA 3’ end processing [55]. A P38 MAPK inhibitor inhibits coagulation, fibrinolysis, and endothelial cell activation [56]. Further research is required to determine the precise impact of the *HDAC9* gene on the coagulation system.

APTT was found to be inversely correlated with active platelets, suggesting that APTT might reflect platelet function [57]. In our GWAS of APTT, we identified three potential candidate genes, and *PRTG* and *BAZ2A* were found to be engaged in platelet activation and thrombosis formation. However, the subfamily of the piggyBac transposable element-derived gene *PGBD2* appears to be limited, and the specific function of *PGBD2* remains unclear.

*PRTG* expression was previously reported to be upregulated in Helicobacter pylori-infected gastric cancer tissues. *PRTG* activates the cGMP/PKG signaling pathway downstream of gastric cancer cells in response to the induction of the epithelial-mesenchymal transition (EMT) transcription factor ZEB1, which has an important role in the progression of gastric cancer [58]. Platelet actin polymerization is required for thrombus stability under flow, and the cGMP/PKG signaling pathway is involved in regulating platelet actin remodeling [59]. Primary hemostasis and arterial thrombosis include platelet activation and aggregation to generate thrombi, and these processes are controlled by intracellular signaling networks [60]. The cGMP/PKG pathway mediates the suppression of platelet aggregation, according to numerous studies [61,62,63].

*BAZ2A*, also called transcription termination factor-1 interacting protein 5 (TIP5), belongs to the bromodomain adjacent to zinc finger proteins (BAZ) family of chromatin remodeling factors, which can have a role in chromatin remodeling, DNA replication, and DNA repair [64]. Previous studies have shown that overexpression of *BAZ2A* can predict tprostate cancer recurrence [65]. In addition, *BAZ2A* can have a regulatory role in hepatocellular carcinoma, cervical cancer, and chronic lymphoblastic leukemia [66,67,68]. MicroRNA (miRNA) is a noncoding RNA that pairs with target messenger RNA (mRNA) in a sequence-specific manner to regulate the expression of target genes [69,70]. One study found that MIR-15a/16-1 at chromosome band 13q14 is downregulated in most patients with chronic lymphocytic leukemia (CLL), and by comparing the expression of MIR-15a/16-1 and computationally predicted MIR-15 a/16-1 target genes in CLL patients and normal controls, *BAZ2A* was identified as the MIR-15a/16-1 specific target gene, and *BAZ2A* was significantly upregulated in CLL patients with MIR-15a/16-1 expression (*p* < 0.05) [68].

In a mouse stroke model, endothelial-targeted deletion of the miR-15a/16-1 cluster bound to complementary sequences in the 3’ untranslated region (3’UTR) of mRNA, inhibiting key proangiogenic factors VEGFA, FGF2, and their receptors VEGFR2 and FGFR1, or cerebral angiogenesis was suppressed after stroke [71]. Yet, *BAZ2A* is a poor indicator of coagulation, and its function in the coagulation cascade requires more research.

Genetic factors are important in determining hemostasis-related phenotypic variation, with APTT and PT showing a significant genetic contribution [72,73]. We performed a heritability analysis for the genes, and the results showed that the heritability of PT and APTT were 0.83 and 0.64, respectively. This supports the idea that genetic variables have a role in APTT and PT results.

Similar studies have been previously reported. For example, an APTT genetic association study in 9719 European Americans and 2799 African Americans identified associations of the *F5, HRG, KNG1, F11, F12,* and *ABO* genes with APTT in African Americans and *KNG1, HRG, F12*, and *ABO* with APTT in European Americans [9]. In another study, the association of *KNG1, HRG, F11, F12*, and *ABO* with APTT was confirmed, and *F7* and *PROCR/EDEM2* were found to significantly affect PT [8]. Moreover, the results of a genome-wide association study of APTT in an elderly population included 488 samples with a mean age of 79.1 years (SD = 0.6), where SNPs on the *F12, KNG1*, and *HRG* genes showed strong associations with APTT, with *F12* rs2731672 (*p* = 2.16 × 10^−30^) showing the strongest association. All of these three genes were associated with the coagulation cascade, and their variants explained 18% of the phenotypic variation in APTT in the study cohort [74]. The aforementioned findings were not confirmed in our investigation, and racial disparities and the health of the included samples probably affect the variations.

The results of our study differ from those obtained in previous works. These differences could be due to the different ethnicity of the population studied, as well as the different inclusion and exclusion criteria used for selecting the study subjects and the sensitivity PT and APTT have towards various preanalytical factors.

The present study has some limitations. First, our study only included a Chinese population, so we did not investigate the impact of ethnicity. Second, as our analyses were based on the PT and APTT of healthy subjects, we need to further investigate the impact of genetic polymorphisms on patients with coagulation disorders or patients taking antithrombotic drugs.

## 5. Conclusions

Our data suggest that the PT and APTT of healthy populations are affected by genetic polymorphisms. *ZNF594* and *ACTN1* variants could be novel genetic markers of PT, while *PRTG* polymorphisms might be associated with APTT levels. The findings could be attributed to ethnic differences. Further research is required to confirm these data.

## Figures and Tables

**Table 1:** Baseline characteristics of the study participants.

Characteristics	Total
N	403
Age (years)	29.5 ± 8.8
Age range (years)	18–60
Female (n)	133 (33.0%)
BMI (kg/m^2^)	22.6 ± 1.8
PT (s)	11.6 ± 1.4
Median PT [range] (s)	11.5 [9.7, 27.8]
APTT (s)	29.6 ± 5.2
Median APTT [range] (s)	28.7 [17.8, 46.4]

**Table 2:** Explaining the heritability of SNPs at the whole genome level.

Heritability of PT			Heritability of APTT		
Source	Variance	SE	Source	Variance	SE
Vg	1.57	0.78	Vg	17.33	10.69
Ve	0.31	0.76	Ve	9.78	10.50
Vp	1.89	0.13	Vp	27.11	1.92
Vg/Vp	0.83	0.40	Vg/Vp	0.64	0.39

Abbreviations: SE, standard error; Vg, genetic variance; Ve, residual variance; Vp, phenotypic variance; Vg/Vp, SNP heritability.

## Data Availability

All data related to the study are shown in the article, and other relevant data can be obtained upon communication with the authors.

## Supplementary Materials

The following supporting information can be downloaded at: https://www.mdpi.com/article/10.3390/genes13101867/s1. Figure S1: PCA principal component analysis diagram; Figure S2: Protein network interaction diagram; Table S1: SNPs that reached *p* < 1 × 10^−5^ from GWAS of PT; Table S2: SNPs that reached *p* < 1 × 10^−5^ from GWAS of APTT.
