# Supplementary material for: Genetic Polymorphisms Associated with Prothrombin Time and Activated Partial Thromboplastin Time in Chinese Healthy Population
Source: Genes (Basel). 2022 Oct 15;13(10):1867. doi: 10.3390/genes13101867 (PMC9602091; doi:10.3390/genes13101867)
Supplement: Supplementary file 1 [file genes-13-01867-s001.zip › supplementary Table S1 SNPs that reached P 1×10^−5^ from GWAS of PT.pdf]

Supplementary Table S1. SNPs that reached P <1×10^{-5} from GWAS of PT

CHR	SNP	MAF	P	GENE	Function
17	rs184838268	0.01061	4.50E-19	ZNF594	missense
15	rs77977805	0.01887	1.88E-13	ATP10A	missense
17	rs144397670	0.01651	6.21E-13	AURKB	intronic
3	rs2271207	0.02005	9.85E-11	SRGAP3	missense
7	rs150273271	0.02005	1.13E-10	IQCE	synonymous
12	rs117348722	0.01061	1.43E-10	FBXW8	synonymous
1	rs55899400	0.01533	8.26E-10	PHGDH	intronic
2	rs72649002	0.01533	8.93E-10	TTN	missense
19	rs200590314	0.01061	9.44E-10	CARM1	intronic
2	rs56372592	0.01533	3.55E-09	AC009948.3	missense
12	rs117876102	0.03302	5.10E-09	USP5	synonymous
12	rs17846773	0.01887	2.46E-08	ABCC9	intronic
20	rs185985671	0.02358	2.74E-08	SIGLEC1	synonymous
22	rs141187399	0.02358	4.48E-08	DNAJB7	missense
4	rs57677641	0.02005	5.76E-08	RNF212	missense
11	rs141576820	0.02948	6.66E-08	RP11-613D13.5	intronic
9	rs2230212	0.05778	7.74E-08	C5	missense
13	rs9566173	0.02712	1.10E-07	FAM48A	intronic
12	rs61736101	0.02712	1.17E-07	KRT73	synonymous
19	rs3764636	0.04835	1.23E-07	CEP89	5'-UTR
12	rs139408145	0.02123	1.29E-07	ABCC9	synonymous
1	rs28914528	0.04835	1.39E-07	ABL2	synonymous
17	rs138594590	0.01533	1.48E-07	TAOK	synonymous
12	rs56207115	0.02241	2.45E-07	KRT72	missense
8	rs143918652	0.02948	3.01E-07	COL14A1	intronic
5	rs77719384	0.0342	4.86E-07	ERBB2IP	synonymous
10	rs2860998	0.08137	5.03E-07	PDLIM1	intronic
10	rs2901833	0.08137	5.03E-07	PDLIM1	intronic
19	rs144304483	0.01179	5.33E-07	KIAA0355	synonymous
10	rs1049961	0.08844	5.57E-07	PDLIM1	3'-UTR
8	rs3829044	0.03066	6.30E-07	COL14A1	intronic
15	rs143745630	0.01179	6.67E-07	MESDC2	3'-UTR
2	rs72648939	0.02358	7.06E-07	TTN	missense
1	rs78881093	0.05307	7.30E-07	AXDND1	intronic
1	rs77282993	0.05307	7.30E-07	AXDND1	missense
1	rs147112292	0.01179	7.64E-07	KIRREL	intronic
10	rs11597005	0.03066	7.80E-07	PKS2L1	intronic
15	rs57365883	0.01297	9.92E-07	MESDC2	stop gained
9	rs2286793	0.03656	1.00E-06	GPR107	intronic
1	rs148354844	0.02241	1.09E-06	DLGAP3	intronic
4	rs3762836	0.01061	1.11E-06	CLOCK	missense

1	rs74475028	0.05425	1.14E-06	AXDND1	intronic
14	rs117434701	0.01061	1.17E-06	HOMEZ	synonymous
14	rs1054200	0.01061	1.17E-06	ACTN1	synonymous
6	rs60429477	0.07665	1.21E-06	C6orf99	intronic
10	rs1049814	0.06958	1.21E-06	PDLIM1	synonymous
2	rs72647856	0.02476	1.26E-06	TTN	intronic
9	rs116919410	0.02241	1.35E-06	DCTN3	intronic
1	rs143062020	0.0283	1.44E-06	PAPPA2	intronic
2	rs562597272	0.01297	1.48E-06	CMPK2	synonymous
3	rs16862731	0.07901	1.52E-06	SERP1	intronic
2	rs137916774	0.01179	1.70E-06	COPS7B	intronic
1	rs545316651	0.02358	1.77E-06	OBSCN	missense
10	rs1049921	0.07311	2.15E-06	PDLIM1	3'-UTR
19	rs2302969	0.1026	2.39E-06	ANKRD27	intronic
1	rs117537503	0.03302	2.48E-06	TRIM11	synonymous
19	rs116968987	0.03538	2.59E-06	DPP9	intronic
17	rs3803764	0.04481	2.97E-06	AZI1	missense
17	rs118178620	0.02241	3.11E-06	ABR	3'-UTR
11	rs147028714	0.01061	3.12E-06	MAP4K2	missense
17	rs77375313	0.03656	3.21E-06	PFAS	intronic
22	rs79004872	0.01297	3.25E-06	THOC5	missense
1	rs146459874	0.01769	3.68E-06	C1ORF127	intronic
7	rs139466052	0.01061	3.71E-06	HDAC9	intronic
8	rs17833992	0.04127	3.75E-06	COL14A1	missense
5	rs1071598	0.01297	3.77E-06	ARSB	missense
17	rs76860463	0.04481	3.82E-06	AZI1	missense
8	rs73329045	0.04019	3.91E-06	COL14A1	intronic
13	rs9568878	0.08608	4.08E-06	STARD13	missense
13	rs438758	0.01533	4.36E-06	COL4A2-AS1	intronic
1	rs193167990	0.01061	4.39E-06	CHIA	5'-UTR
8	rs3815235	0.03066	4.59E-06	RP11-666I19.2	intronic
12	rs12580578	0.07075	4.88E-06	DNAH10	missense
12	rs7977449	0.07075	4.88E-06	DNAH10	missense
12	rs11834289	0.07075	4.88E-06	DNAH10	missense
12	rs61732737	0.07075	4.88E-06	DNAH10	missense
9	rs148657560	0.01061	5.29E-06	CACFD1	synonymous
10	rs138177179	0.02712	5.39E-06	11kb 5' of RP11-14C22.6	intronic
22	rs6971	0.02123	5.47E-06	TSPO	missense
2	rs375873175	0.01061	5.56E-06	FMNL2	intronic
19	rs3745382	0.1215	5.70E-06	AC010336.2	synonymous
3	rs190629132	0.01297	5.87E-06	MKRN2	intronic
16	rs184754710	0.01061	6.18E-06	ADAD2	missense
1	rs144266216	0.01651	6.27E-06	MASH4	intronic

19	rs188290430	0.01533	6.32E-06	ANGPTL6	intronic
8	rs74885248	0.01179	6.49E-06	DOCK5	missense
22	rs188743876	0.01179	6.51E-06	MAFF	3'-UTR
22	rs61740613	0.01179	6.70E-06	THOC5	missense
22	rs147790737	0.01179	6.70E-06	NIPSNAP1	intronic
19	rs79388179	0.02358	6.84E-06	CLECC4G	3'-UTR
1	rs3754204	0.02948	7.09E-06	HOOK1	intronic
10	rs2862954	0.07665	7.11E-06	ERLIN1	missense
12	rs7969440	0.07311	7.47E-06	DNAH10	synonymous
12	rs192841505	0.01887	7.54E-06	CCDC53	intronic
10	rs12784396	0.07547	7.56E-06	CWF19L1	5'-UTR
12	rs7969937	0.07193	7.69E-06	DNAH10	missense
12	rs74723106	0.07193	7.69E-06	DNAH10	intronic
3	rs139264479	0.01415	7.79E-06	LIPH	synonymous
3	rs376952206	0.02005	7.93E-06	WNT5A	intronic
8	rs74196658	0.01887	8.07E-06	TM2D2	missense
3	rs3814404	0.02005	8.11E-06	BOC	missense
8	rs77866076	0.01533	8.47E-06	KIAA1875	missense
1	rs188577493	0.01061	8.63E-06	SNX27	intronic
17	rs191977923	0.01651	8.99E-06	EVPL	missense
2	rs202055795	0.01179	9.42E-06	TTC31	missense
