# Supplementary material for: Genetic Polymorphisms Associated with Prothrombin Time and Activated Partial Thromboplastin Time in Chinese Healthy Population
Source: Genes (Basel). 2022 Oct 15;13(10):1867. doi: 10.3390/genes13101867 (PMC9602091; doi:10.3390/genes13101867)
Supplement: Supplementary file 1 [file genes-13-01867-s001.zip › supplementary Table S2 SNPs that reached P 1×10^−5^ from GWAS of APTT.pdf]

Supplementary Table S2 SNPs that reached P <1×10^{-5} from GWAS of APTT

**CHR**	**SNP**	**BP**	**MAF**	**P**	**GENE**	**Function**
1	rs75935520	249211729	0.01061	4.49E-06	PGBD2	missense
12	rs199970765	56994493	0.01533	5.69E-06	BAZ2A	missense
15	rs80064850	55974495	0.01297	8.69E-06	PRTG	intronic
