# Supplementary figures and images for: Genetic Polymorphisms Associated with Prothrombin Time and Activated Partial Thromboplastin Time in Chinese Healthy Population
Source: Genes (Basel). 2022 Oct 15;13(10):1867. doi: 10.3390/genes13101867 (PMC9602091; doi:10.3390/genes13101867)

**Supplementary Figure S1** PCA principal component analysis diagram

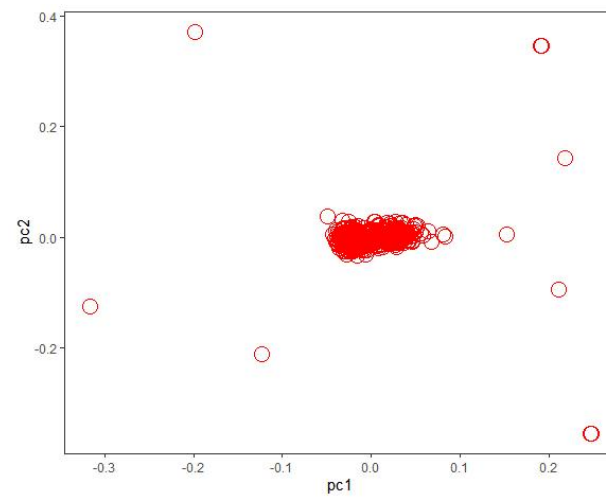

Supplement: Supplementary file 1 [file genes-13-01867-s001.zip › supplementary Figure 1 PCA principal component analysis diagram.pdf]

**Supplementary Figure S2 Protein network interaction diagram**

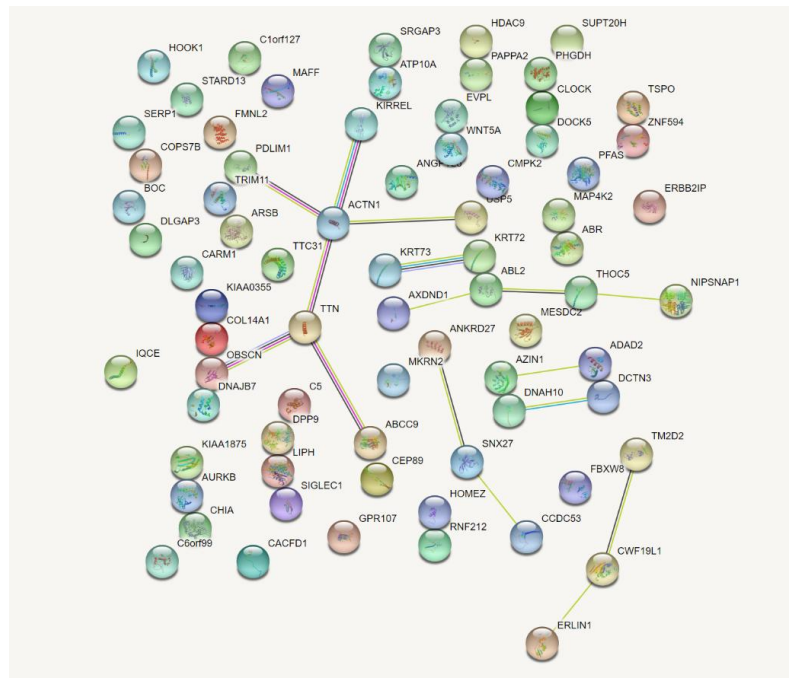

Supplement: Supplementary file 1 [file genes-13-01867-s001.zip › supplementary Figure 2 Protein network interaction diagram.pdf]
